# Elucidating the Dehydration Pathways of K_2_CO_3_·1.5H_2_O

**DOI:** 10.1021/acs.cgd.3c01484

**Published:** 2024-03-04

**Authors:** Joey Aarts, Natalia Mazur, Hartmut R. Fischer, Olaf C. G. Adan, Henk P. Huinink

**Affiliations:** † Eindhoven Institute of Renewable Energy Systems, Eindhoven University of Technology, PO Box 513, Eindhoven 5600 MB, The Netherlands; ‡ Transport in Permeable Media group, Department of Applied Physics, Eindhoven University of Technology, PO Box 513, Eindhoven 5600 MB, The Netherlands; § TNO Materials Solution, PO Box 6235, High Tech Campus 25, Eindhoven 5600 HE, The Netherlands

## Abstract

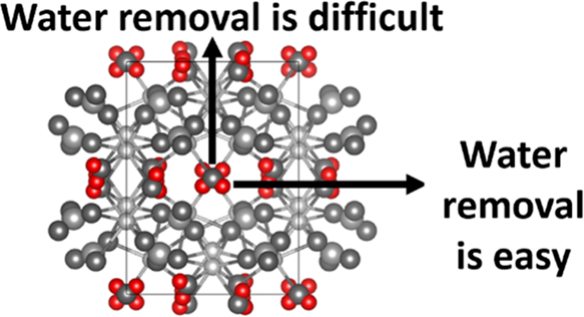

Potassium carbonate sesquihydrate has previously been identified as a promising material for thermochemical energy storage. The hydration and cyclic behavior have been extensively studied in the literature, but detailed investigation into the different processes occurring during dehydration is lacking. In this work, a systematic investigation into the different dehydration steps is conducted. It is found that at higher temperatures, dehydration of pristine material occurs as a single process since water removal from the pristine crystals is difficult. After a single cycle, due to morphological changes, dehydration now occurs as two processes, starting at lower temperatures. The morphological changes open new pathways for water removal at the newly generated edges, corners, and steps of the crystal surface. The observations from this work may contribute to material design as they elucidate the relation between material structure and behavior.

## Abstract 2

Potassium carbonate sesquihydrate has previously been identified as a promising material for thermochemical energy storage.

## 1 Introduction

In The Netherlands, at least half of the total energy consumption originates from heating, which is still primarily generated from fossil or nonsustainable sources.^1^ On the other side, a major part of future energy production will be covered by renewables. However, sources such as wind or solar energy provide inconsistent output. Consequently, to store energy during abundant times until demanding times, an efficient storage method is required.

A suitable method of storing energy is thermochemical energy storage. Potassium carbonate has been proposed as a candidate for thermochemical energy storage, based on several key performance indicators (KPIs). These KPIs are energy density, safety, price, and abundance.^2^ The reversible hydration and dehydration reaction of potassium carbonate, producing heat *Q*, proceeds as
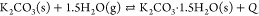
1Due to the promising nature of K_2_CO_3,_ it has been researched extensively over the past few years. Hydration/dehydration kinetics and the cyclic performance of potassium carbonate have been well studied. The hydration and dehydration kinetics of powder have been studied by Fisher et al. and Gaeini et al., and the hydration kinetics of particles have been studied by Aarts et al.^3−5^ A detailed study on the dehydration of precycled potassium carbonate was performed by Mazur et al.^6^ Cyclic testing of powder was performed by Sögütoglu et al., whereas cyclic testing of larger particles has been performed by Beving et al. and Aarts et al.^7−9^ Testing of bed performance was done by Mahmoudi et al.,^10^ Houben et al.,^11^ and Raemaekers et al.^12^ In addition, the effect of CO_2_ on the performance of K_2_CO_3_ was investigated.^13^

Detailed kinetic analysis led to the observation of a metastable zone for hydration and dehydration. As a result, the reaction kinetics is extremely slow when crossing the equilibrium line, while remaining inside the metastable zone.^14−16^ This metastable behavior originates from a nucleation barrier at low supersaturations. Attempts were made to increase the kinetics and decrease the width of the metastable zone of potassium carbonate by doping with organic or inorganic dopants.^17,18^

The focus of the literature available is on hydration (mechanisms), whereas only a few studies have yet been performed on the dehydration processes at play. Deshpande et al. suggested that potassium carbonate dehydrates in two steps. First 0.5 mol of water is removed forming the monohydrate followed by removing the last 1 mol of water forming the anhydrous material.^19^ A similar suggestion is made in recent literature based on the presence of two different types of water molecules in the crystal lattice.^6^ Furthermore, the literature refers to precycled or preconditioned materials, large particle sizes, or large sample sizes, which all may influence the dehydration behavior.^4,6,20^

In addition to thermochemical materials, the dehydration of organic (pharmaceutical) hydrates is also investigated in literature.^21−23^ However, these dehydration studies do not focus on a higher number of dehydration and rehydration cycles. This, however, is critical in terms of thermal energy storage.

This study aims to elucidate the exact mechanism of potassium carbonate dehydration. In view of application, the dehydration behavior is just as important as the hydration behavior as the dehydration behavior determines how charging of a heat battery will proceed. Therefore, if indeed intermediate dehydration steps are present, then the effect on charging should be investigated. In addition to that, the dehydration of pristine material should be investigated, since in a real application precycling is not desired, and possible difficulties during early cycles should be mitigated in some way.

Therefore, first, the observations during dehydration of potassium carbonate during various cycles are discussed, followed by a detailed analysis of the possible origins of the observed effects. Afterward, all observations are linked to the crystal structure of single crystals, which are then translated to powder samples. The insights from this work could provide insight into the dehydration mechanisms of other salt hydrates as well and may serve as input for thermochemical material design.

## 2 Materials and Methods

### 2.1 Materials

Potassium carbonate sesquihydrate and potassium bicarbonate (both provided by Evonik Functional Solutions GmbH, > 99% purity) were milled using a Fritsch planetary ball mill and sieved into a 50–164 μm fraction. The material was used without any further modification and stored in airtight containers.

### 2.2 Thermogravimetric Analysis (TGA)

TGA was performed on a Mettler Toledo TGA/DSC3+. The TGA setup is equipped with a home-built humidifier generating different water vapor pressures by mixing a completely wet and completely dry nitrogen gas stream (300 mL/min). This allows one to perform cyclic studies on powder without removing the samples from the TGA device. For all measurements, hydrated potassium carbonate was placed in 40 μL Mettler Toledo standard aluminum pans without a lid. In all experiments, the sample was first kept at the hydration temperature for 2 h to confirm the starting material was fully hydrated.

Relative humidity (RH) calibration was done by checking the deliquescence of various salts at 25 °C (LiCl·H_2_O, K_2_CO_3_·1.5H_2_O, MgCl_2_·6H_2_O and Mg(NO_3_)_2_·6H_2_O). Temperature calibration was performed by checking the melting points of three metals (indium, zinc, and lead). Calibration is described in more detail in Sögütoglu et al.^7^

### 2.3 Differential Scanning Calorimetry (DSC)

DSC measurements were conducted on a Mettler Toledo DSC822e with a 1 K/min heating rate under a nitrogen atmosphere. For all measurements, hydrated potassium carbonate or potassium bicarbonate was placed in 40 μL Mettler Toledo standard aluminum pans without a lid. As a reference pan, a similar pan without a lid was used. Temperature and heat flow calibration was performed using naphthalene, benzophenone, benzoic acid, indium, and zinc.

### 2.4 Powder X-ray Diffraction (PXRD)

Powder X-ray diffraction (PXRD) was performed using a Rigaku Mini-Flex diffractometer in continuous scan mode with a divergent slit of 0.625° using a D/teX Ultra2 detector, using Cu Kα radiation and a Kβ filter. The diffractometer is equipped with an Anton Paar BTS500 benchtop heating stage with an attached home-built humidifier. The data were gathered between 10 and 50° 2θ with step sizes of 0.01° and a scanning speed of 5°/min. Measurements were performed under isobaric water vapor conditions of 5 mbar in air. In situ dehydration and rehydration were performed at 160 and 25 °C, respectively.

The primary scattering domain size was calculated with the PDXL software by Rigaku. For the calculations, the full width at half-maximum of a peak and a Scherrer constant of 0.94 were used. The averages and standard deviations are based on the entire powder pattern.

### 2.5 Neutron Imaging

Neutron imaging was performed at the Paul Scherrer Institute (PSI) in Switzerland at the ICON beamline. Neutrons are generated using a spallation neutron source at PSI after which they are moderated using liquid deuterium at 25 K. For a detailed instrumental description, the reader is referred to Kaestner et al.^24^ For analysis, the samples are placed within aluminum containers which are transparent for the used neutrons. This was done to prevent interaction between the surrounding atmosphere and the sample.

### 2.6 Optical Microscopy of Single Crystal Dehydration

K_2_CO_3_ single crystals were grown from solution under room conditions. Potassium carbonate sesquihydrate (provided by Evonik Functional Solutions GmbH) was dissolved in deionized water to form a concentrated salt solution. The beaker with that solution was covered with a watch glass to limit the evaporation rate and left on the fume hood bench in the lab. After several days, single crystals have formed in the solution. Selected crystals were taken out of the solution, dabbed dry with tissue, and stored in an airtight container for later use.

The dehydration experiments were performed using Zeiss SteREO Discovery V20 microscope equipped with Linkam THMS600-H Hot stage connected to Linkam RH95 Humidity Controller, Dehydration was conducted at 105 °C and 0 mbar water vapor pressure. Compressed air was used to remove generated water vapor and maintain the desired humidity inside the chamber. The apparatus is described in detail in Beving et al.^8^

### 2.7 Scanning Electron Microscopy (SEM)

SEM analysis on single crystals and powder were performed on a FEI Quanta 600 using high vacuum (<10^–4^ mbar). Partially dehydrated crystals were prepared in a lab oven. The crystals were dehydrated at 130 °C for 6 h shortly before SEM imaging. Prior to analysis of single crystals, the crystal was cleaved through the middle by applying pressure with a razor blade. Crystals were fixed to the SEM stub with carbon tape and imaged right after cleaving.

## 3 Results and Discussion

### 3.1 Two Dehydration Processes

**Figure 1:**
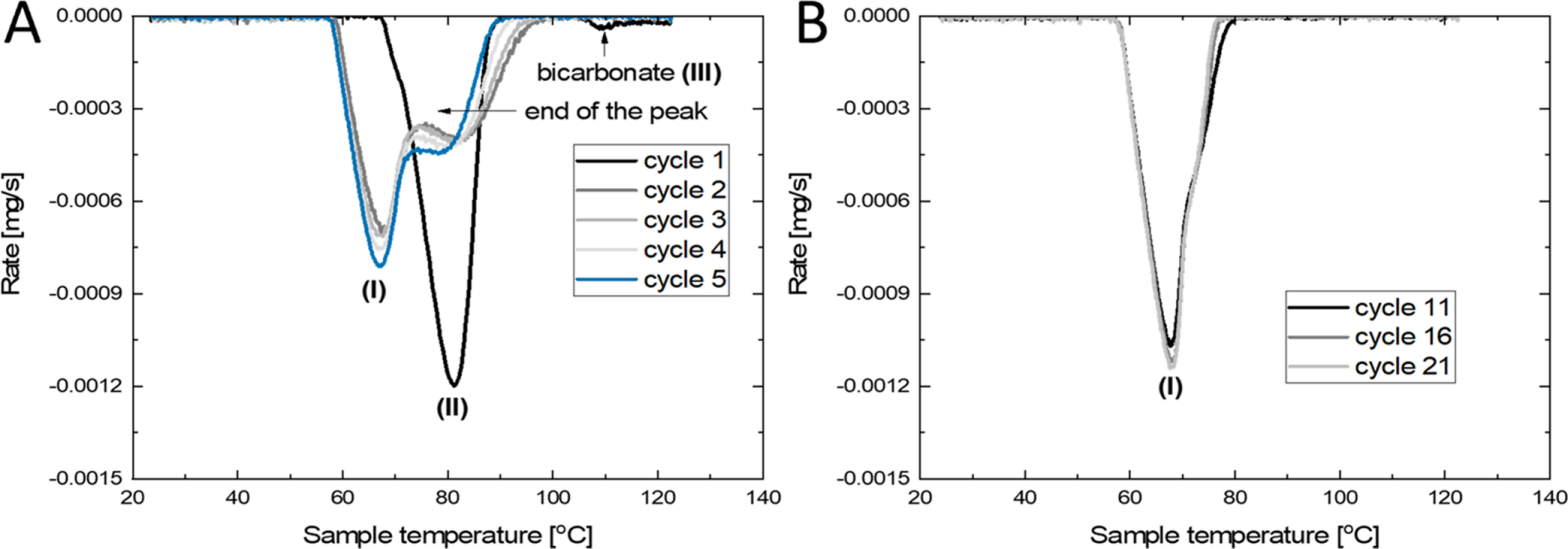
TGA cyclic rate curves versus sample temperature for the first 5 dehydrations (A) and dehydrations 11, 16, and 21 (B). Used conditions are 5 mbar of water vapor with set temperature cyclic ramping between 25 and 130 °C at 1 K/min. A negative rate indicates mass loss. The different processes are denoted by (I), (II), and (III).

The cyclic hydration and dehydration behavior were characterized using fully hydrated, uncycled, 50–164 μm potassium carbonate powder. Measurements were conducted using isobaric conditions (5, 10, and 15 mbar) with varying temperatures. An example measurement sequence for 5 mbar can be found in Supporting Information S1.1. The rate was calculated by differentiating the weight versus the time curve. As an example, the resulting cyclic rate curves for 5 dehydrations at 5 mbar are given in Figure 1A.

The 5 hydration rate curves show similar behavior for every cycle. Upon decreasing the temperature, the hydrate rate increases until it drops to zero when the hydration reaction is complete. These observations match with the available literature.^14^ The hydration curves can be found in Supporting Information S1.2.

For this study, the dehydration results are of interest. The first dehydration shows a single peak with a maximum of around 80 °C corresponding to the dehydration reaction (called process II), followed by a tiny second peak around 110 °C which is attributed to potassium bicarbonate decomposition (called process III).^13^ The second and later dehydration events show mass loss at a lower temperature (called process I) compared with the first cycles. Instead of being composed of a single peak, bimodal peaks are now observed, of which the end of the peak shifts toward lower temperature with cycling, merging into a single peak after 10 cycles.

To identify whether a stable dehydration behavior would appear at higher cycles, a sample at 5 mbar of water vapor was cycled for up to 21 cycles. The rate curves for dehydration are shown in Figure 1B. It is observed that at higher cycles, the second dehydration peak (process II) has merged into the first peak (process I), with a small remaining shoulder, after which constant dehydration behavior with cycling is observed. This indicates the presence of two processes, of which the rates are increasing with cycling, causing the end of the peak to shift toward lower temperatures.

Additionally, it is observed that the maximum rate of process I compared with dehydration process II increases with cycling. After dehydration 11, no significant increase in the rate is observed between dehydrations 11, 16, and 21. This matches with observations made by Mazur who observed that until cycle 10, due to structural reorganization, the rate increased significantly with each cycle whereas after cycle 10 the rate increase with cycling decreased.^25^ Mazur et al. have shown using SEM imaging how the porosity visibly increases after cycling.^6^ The figure of powder after 11 dehydration events in Supporting Information S1.3. shows a similar structure.

**Figure 2:**
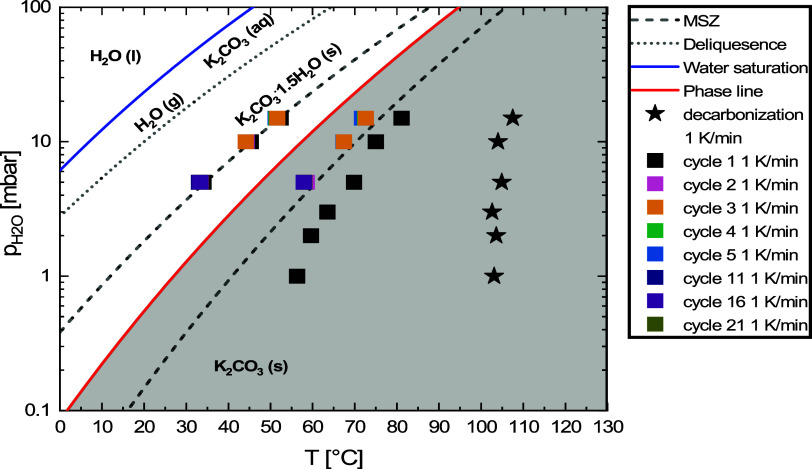
Hydration, dehydration, and decarbonization onset points. Used conditions are 15 mbar water vapor with set temperature cyclic ramping between 40 and 130 °C, 10 and 5 mbar water vapor with set temperature cyclic ramping between 25 and 130 °C, and 3, 2, and 1 mbar water vapor with set temperature ramping between 20 and 130 °C. All temperature ramp rates were set at 1 K/min.

Using the rate curves, the onset points for hydration, dehydration, and decarbonization can be determined, for which the exact procedure is described in Supporting Information S1.4. The resulting onset points for various isobaric conditions are given in Figure 2. Note that for 3, 2, and 1 mbar of water vapor only the first dehydration and decarbonization (decomposition of bicarbonate) onset points are given. This was due to minimum temperature constraints inside the TGA furnace, and rehydration could not be performed outside of the hydration metastable zone at these water vapor pressures.

The onset points in Figure 2 are directly compared to available literature data on metastable zones published by Sögütoglu et al.^14^ It is observed that all hydration onset points match well with the already known hydration metastable zone, and the first cycle of decarbonization is found to be independent of the supplied water vapor pressure within the investigated regime. For dehydration event 2 and onward (colored squares) the onset points also match with the earlier published data.^14^ However, a difference is observed for the first dehydration event (black squares). The dehydration onset points of the first dehydration event are found at higher temperatures for the dehydration of pristine material.

The reason for the difference for the first cycle results from different measurement approaches. In the work of Sögütoglu et al., the hydrate material is first fully dehydrated prior to the measurements. As a result, the first cycles of dehydration and decarbonization are not reported in that study.

### 3.2 Separating the Dehydration Processes

**Figure 3:**
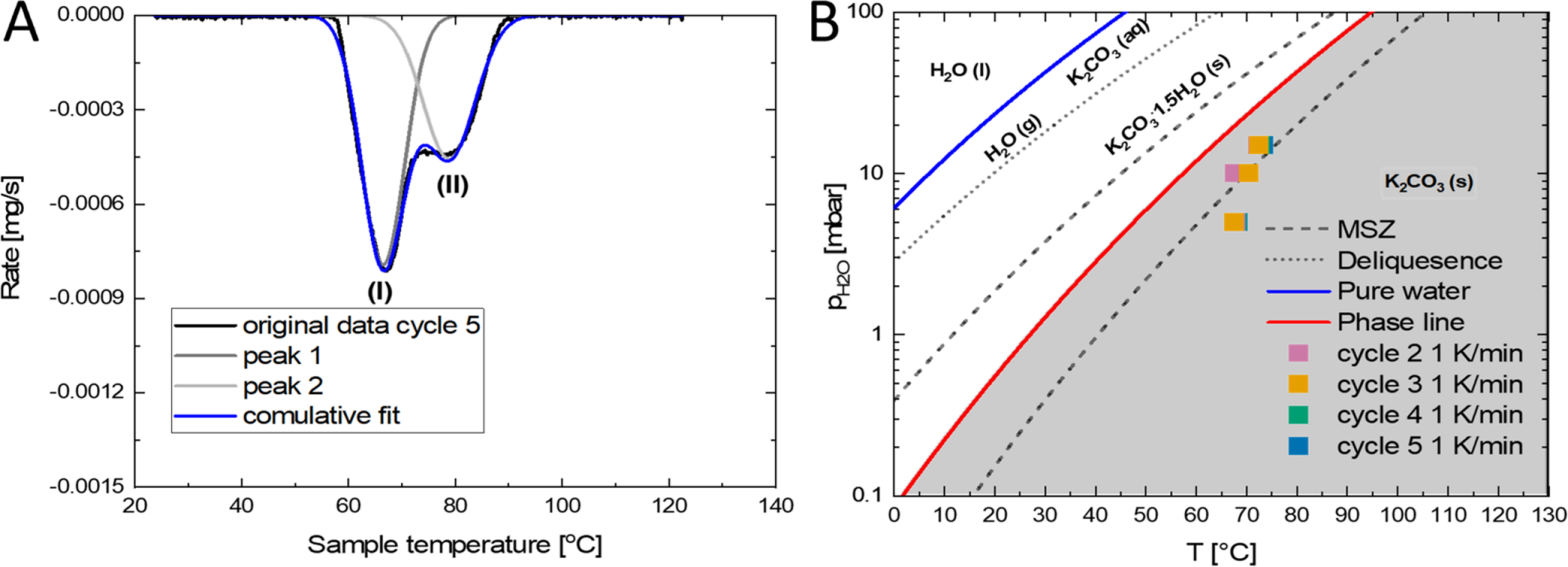
Gaussian peak deconvolution of the 5th dehydration at 5 mbar of water vapor pressure (A) and the onset points for the second peak (process II) (B). Used conditions are 15 mbar of water vapor with set temperature cyclic ramping between 40 and 130 °C and 10 and 5 mbar of water vapor with set temperature cyclic ramping between 25 and 130 °C. All temperature ramp rates were set at 1 K/min. A negative rate indicates mass loss. MSZ denotes the metastable zone. The different processes are denoted by (I) and (II).

To further understand the double peak behavior observed during dehydration 2 and onward, peak deconvolution was performed to find the onset point for the second peak (process II) from dehydration event 2 and onward. An example of the Gaussian deconvolution performed on the fifth dehydration at 5 mbar of water vapor pressure is given in Figure 3A.

The deconvolution of peaks can then be used to find the onset point for the second peak (process II) for dehydration event 2 and onward according to the same procedure as described in Supporting Information S1.4. The resulting onset points are listed in Figure 3.

It is observed that the onset points for the second peak (process II) do not vary significantly with cycling. At higher water vapor pressures (10 and 15 mbar) the onset points are closer to the metastable zone line proposed by Sögütoglu et al.^14^ However, at lower vapor pressure (5 mbar) the onset points seem to deviate toward higher temperatures. It must be noted however that in the work of Sögütoglu et al. the lowest measured vapor pressure is 7 mbar and values below suffer from larger uncertainty.^14^ For comparison, values for the dehydration onset points from Figures 2 and 3B are combined into tabular format in Table 1.

**Table 1:** Summary of Onset Points from Figures 2 and 3B

dehydration	pressure [mbar]	onset peak 1 [°C]	onset peak 2 [°C]
**1**	5	69.8	
	10	75.0	
	15	81.1	
**2**	5	58.6	67.9
	10	67.4	67.7
	15	72.6	72.2
**3**	5	58.0	67.5
	10	67.4	70.4
	15	72.6	72.5
**4**	5	58.1	68.2
	10	67.4	68.8
	15	72.4	72.8
**5**	5	57.9	68.3
	10	67.3	68.5
	15	71.7	73.4

### 3.3 Effect of Possible Impurities on the Dehydration Behavior

To determine the origin of the double peak behavior, a series of tests was performed. First, the effect of the possible impurities was investigated. Potassium carbonate can form bicarbonate in the presence of water vapor and CO_2_ gas.^13,26^ Moreover, TGA experiments in this work show that the starting material contains some (1–2%) bicarbonate. Since the hydrated powder is stored in an airtight container but not inert atmosphere, there could be possible traces of the recently proposed double salt.^13^

**Figure 4:**
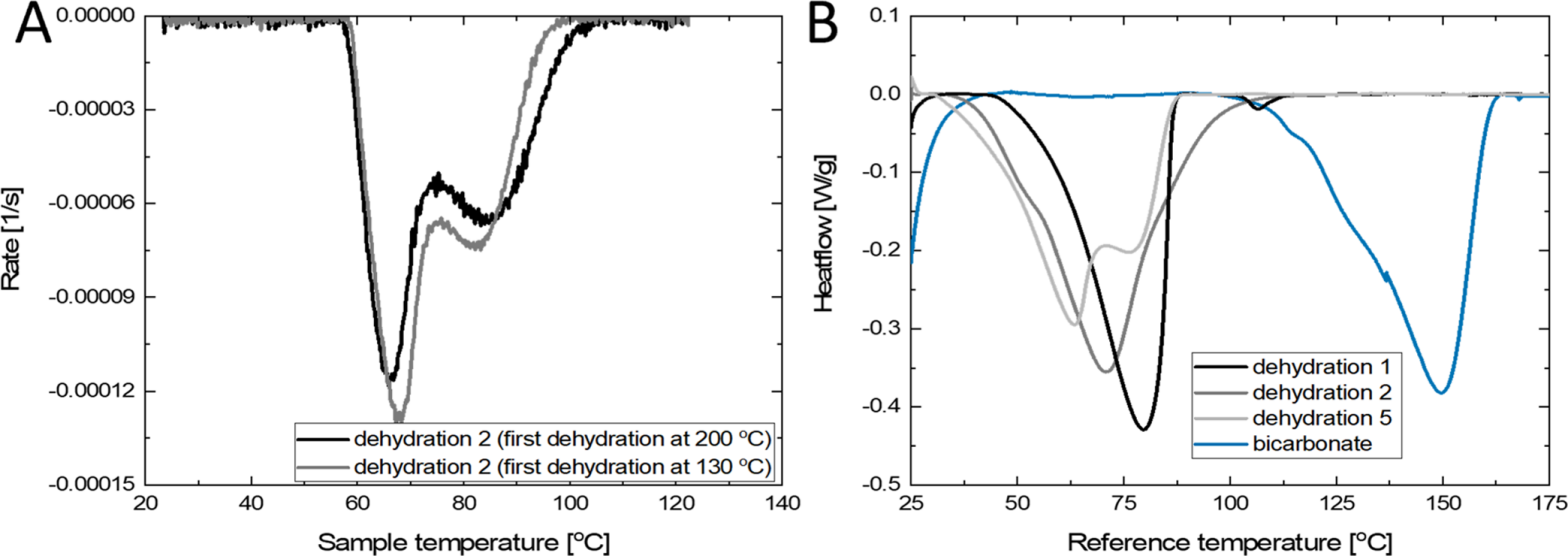
TGA rate curves versus sample temperature for dehydration 2 (A). The black and gray lines were dehydrated at 200 and 130 °C during dehydration 1, respectively. Rehydration and the second dehydration were performed as described before. DSC thermograms for various dehydrations and decarbonization of pure bicarbonate (blue line) (B). The samples were cycled inside the TGA as described before being transferred to the DSC. The used scan rate was 1 K/min under a pure N_2_ atmosphere (no water vapor).

To exclude the effect of residual bicarbonate or double salt, dehydration was performed at a higher temperature. A sample at 5 mbar underwent dehydration at 200 °C for 4 h to ensure all impurities are removed. Next, the sample was rehydrated and dehydrated, as described before. The second dehydration of the sample, which was first dehydrated at 200 °C, was compared to the second dehydration from Figure 1A. The rates were rescaled to the sample mass to exclude the effect of weight difference. The resulting rate curves are given in Figure 4A. It is found that both samples show similar dehydration behavior during dehydration 2. Both samples show the same onset point and double peak behavior.

In addition, DSC measurements were performed on uncycled and cycled hydrated materials as well as a fresh bicarbonate sample. The cycled hydrated potassium carbonate was prepared inside the TGA at 5 mbar as described before, after which the sample was quickly transferred to the DSC. The DSC measurements were conducted under a pure N_2_ atmosphere (no water vapor) and are given in Figure 4B.

The DSC thermograms show similar behavior as that observed in TGA. The first dehydration shows a single peak followed by a decarbonization peak, which is found at the same temperature as the pure bicarbonate decarbonization peak. Dehydration 2 shows a shoulder at the low-temperature region, and dehydration 5 shows the same double peak behavior without the presence of impurity peaks. Therefore, it is concluded that there is no effect of bicarbonate impurities.

### 3.4 Effect of Scanning Rate on the Dehydration Onset

**Figure 5:**
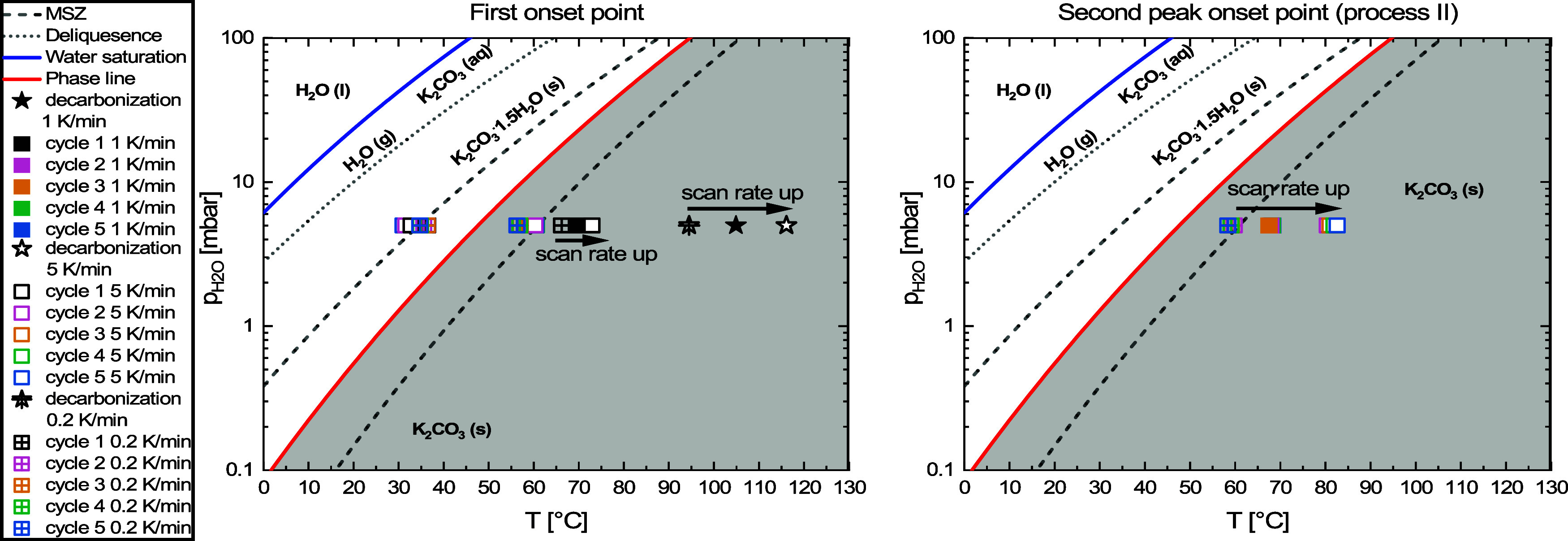
Onset points for hydration, dehydration, and decarbonization at various scan rates (0.2 1, and 5 K/min). Used conditions are 5 mbar of water vapor with set temperature cyclic ramping between 25 and 130 °C.

Next, the scan rate was changed to investigate the effect on the positions of the onset points. A high scan rate dependency indicates that the processes are more nucleation limited, whereas a low scan rate dependency indicates the opposite. A fast (5 K/min) and slow (0.2 K/min) scan rate were added, and the determination of the onset points was done in a similar way as described in Supporting Information S1.4. The first onset points and the second peak onset points are listed in Figure 5.

Obviously, the hydration and dehydration onset points (from cycle 2 onward) are slightly affected by changing the scanning rate, whereas the onset for dehydration 1 (process II) is affected more. The two onsets that are most affected are the decarbonization and onset point of the second peak (process II). This indicates that the first dehydration event and the onset for the second peak (both process II) are more nucleation limited, and the nucleation rate is slow. Hydration and the onset for dehydration from cycle 2 onward (process I) are less affected by the scan rate, and nucleation is therefore faster in these processes. The values from Figure 5 are summarized in Table 2.

**Table 2:** Summary of Dehydration Onset Points from Figure 5

dehydration	scan rate [K/min]	onset peak 1 [°C]	onset peak 2 [°C]
**1**	0.2	66.2	
	1	69.8	
	5	72.9	
**2**	0.2	56.8	59.8
	1	58.6	67.9
	5	60.3	80.4
**3**	0.2	57.0	59.2
	1	58.0	67.5
	5	60.4	81.0
**4**	0.2	56.9	59.1
	1	58.1	68.2
	5	60.6	81.9
**5**	0.2	56.3	58.4
	1	57.9	68.3
	5	60.5	82.6

### 3.5 Apparent Activation Energies for Both Processes

The observation that there are two different processes that differ in their nucleation speed is further illustrated when calculating the apparent activation energy versus the dehydration conversion. The apparent activation energy can be calculated from various isothermal and isobaric measurements according to equations^27^
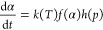
2with
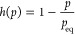
3and
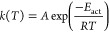
4Here α [-], *t* [s], *E*_act_ [J·mol^–1^], *R* [J·mol^–1^·K^–1^], *T* [K], *p* [Pa], and *p*_eq_ [Pa] represent the conversion, time, apparent activation energy, universal gas constant, temperature, applied water vapor pressure, and equilibrium water vapor pressure, respectively. Furthermore, *f*(α) is the function describing conversion progress, and *A* is the pre-exponential factor within the Arrhenius equation. Vapor pressures are chosen far from equilibrium so that the term *h*(p) can be assumed to be unity.

**Figure 6:**
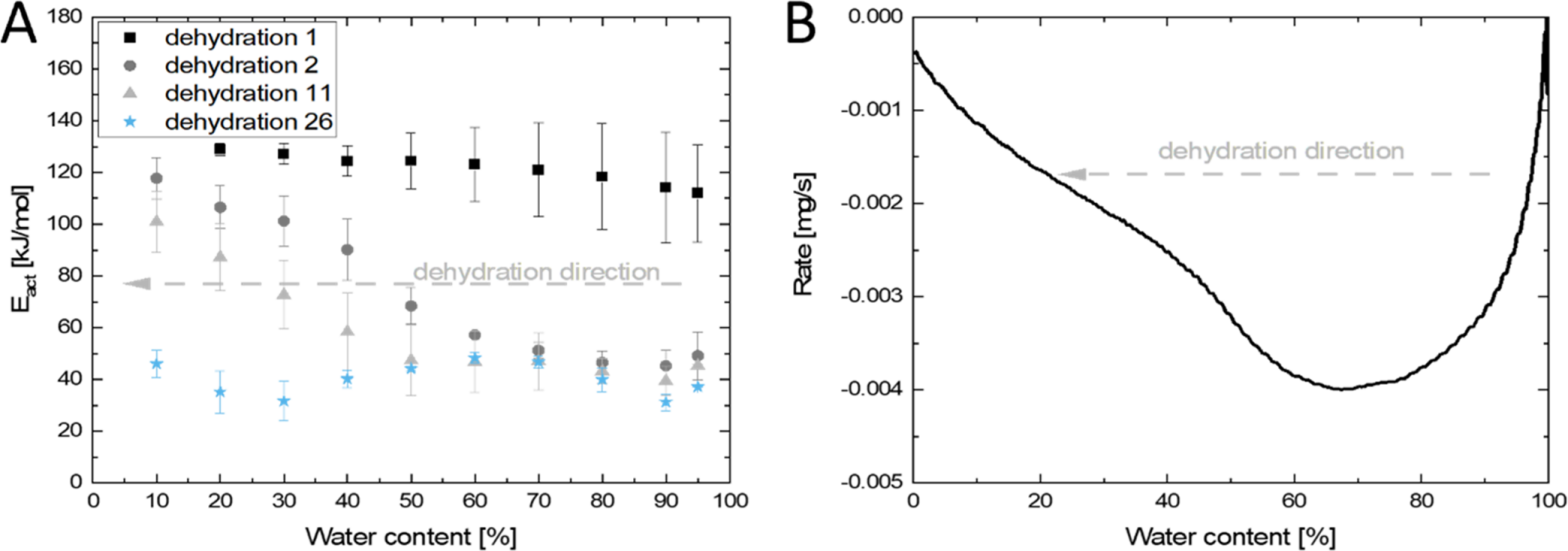
Apparent activation energy vs water content for dehydration 1, 2, 11, and 26 at isobaric water vapor pressure of 5 mbar (A). The apparent activation energy is obtained at 3 temperatures (75, 90, and 100 °C). The dehydration direction is denoted by the gray dashed arrow. Dehydration rate for the second dehydration event versus water content for a sample dehydrated at 100 °C and 5 mbar (B).

When plotting ln(dα/d*t*) versus 1/*RT* at fixed water content and vapor pressure a linear fit can be used to extract *E*_act_ as the slope at the chosen water content. The apparent activation energy versus the water content for dehydration events 1 and 2 at 5 mbar is given in Figure 6A.

For the first dehydration, a constant apparent activation energy is observed around 120–135 kJ/mol. For the second dehydration, an initial apparent activation energy of 45–60 kJ/mol is observed, which increases at around 75% water content toward the value observed for dehydration 1. This matches with Figure 6B where the dehydration rate decreases around 75% water loading and the second process starts.

During dehydration 11 the increase in apparent activation energy is found at lower water content, matching with the merging of peaks in Figure 1. This illustrates that indeed process II is becoming less dominant compared to process I. After 26 dehydrations only a single activation energy is found matching with the observation that indeed the dehydration is occurring only through process I.

The constant apparent activation energy for dehydration 1 matches the observation that only a single process (peak) occurs. The found values are in a similar range (80–120 kJ/mol) observed by Mazur et al.^6^ The lower initial apparent activation energies for dehydration events 2, 11, and 26 are similar, which indicates that at higher numbers of dehydration the apparent activation energy of process I does not decrease further.

### 3.6 Microscopy and Single Crystal Dehydration

**Figure 7:**
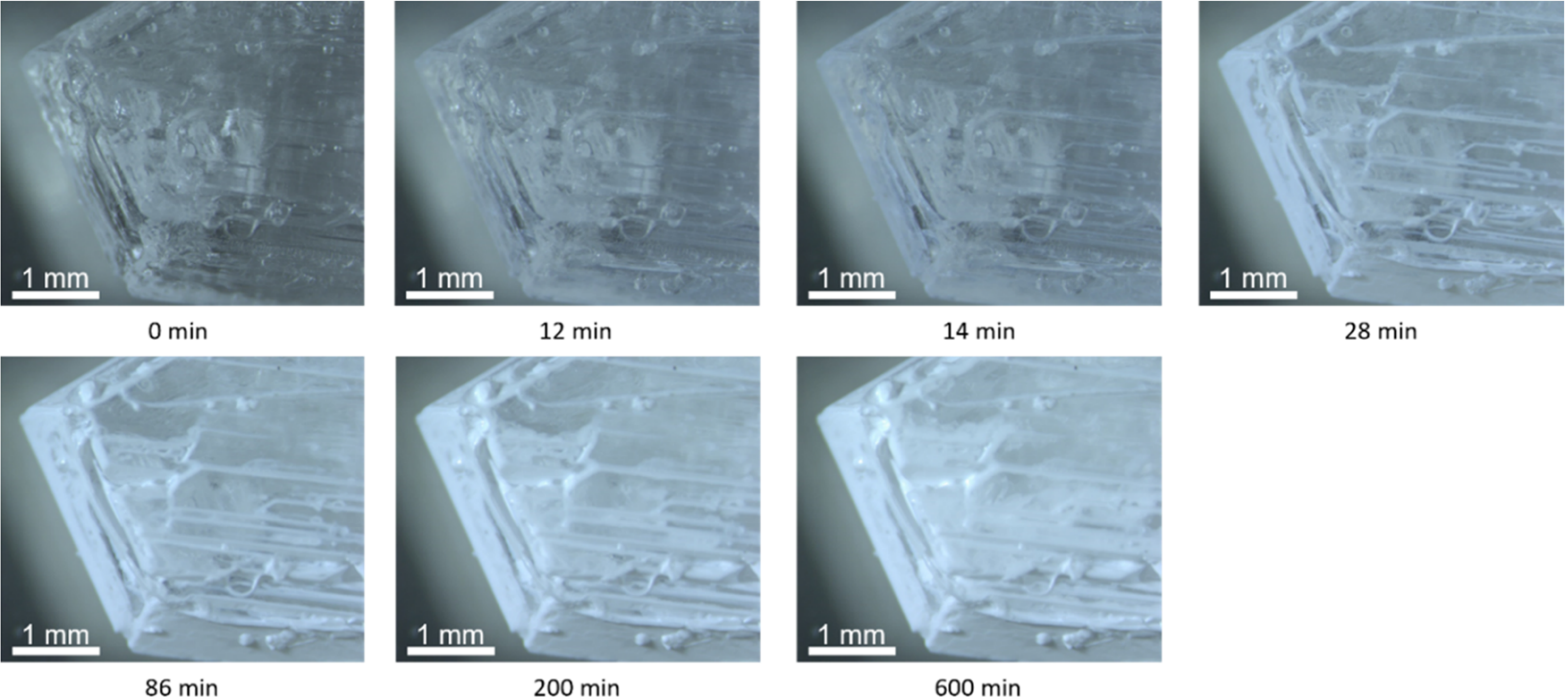
Dehydration under isothermal conditions of 105 °C and 0 mbar vapor pressure. Dehydration is visible as the transition from transparent to opaque (white).

The dehydration of a single crystal was studied to elucidate the different dehydration processes. Images were taken at different times during the dehydration process at 105 °C and 0 mbar of water vapor, and the selected images are displayed in Figure 7.

The sesquihydrate single crystal starts completely transparent (0 min). After 28 min, the edges and steps of the single crystal visibly start to dehydrate, resulting in a white outline. At 200 and 600 min, after the edges have dehydrated, the planes start to visibly dehydrate changing to a white color.

**Figure 8:**
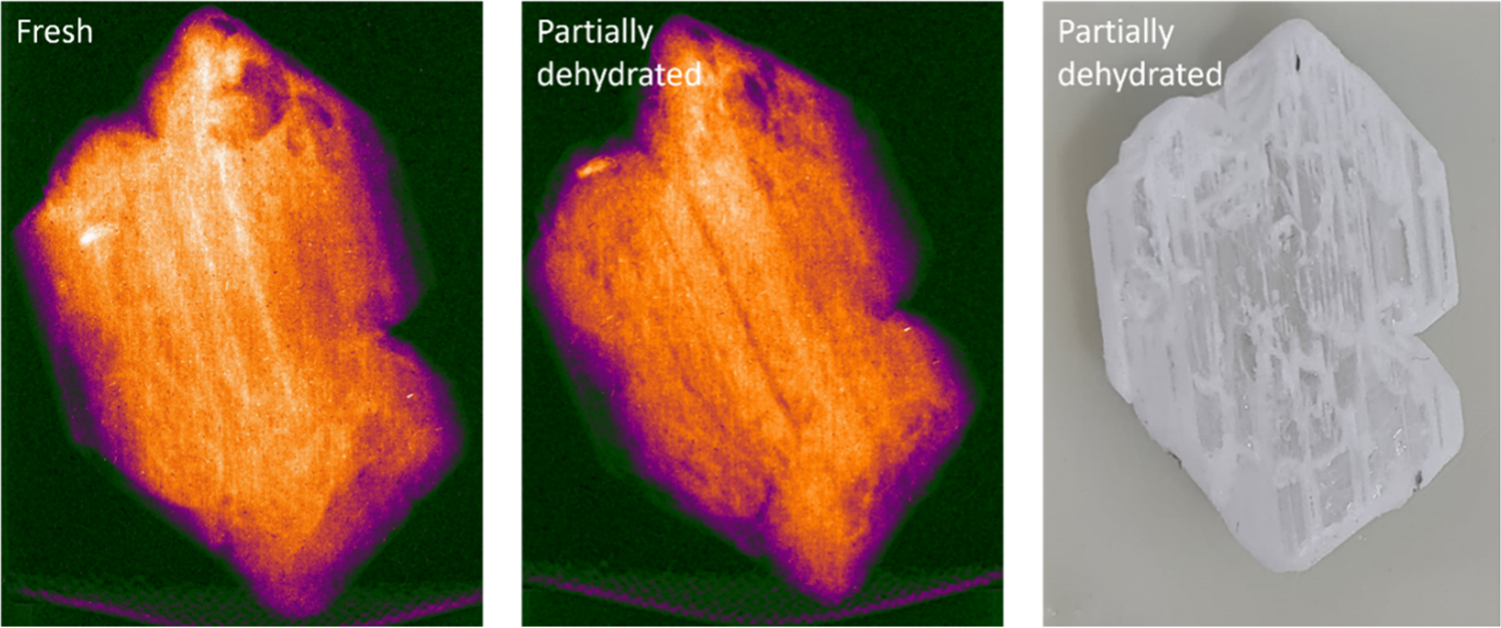
Neutron radiograph of a fresh K_2_CO_3_ sesquihydrate single crystal and the same crystal partially dehydrated in an oven at 105 °C for 6 h. Bright spots in the neutron image indicate a low neutron transmission. An optical image of the same partially dehydrated crystal after neutron imaging has been added as a reference.

An attempt to visualize the dehydration behavior was made by using neutron imaging (Figure 8). When the neutron image of the fresh and partially dehydrated sample is compared, the disappearance of lines is visible. It is hypothesized that these lines correspond to the edges and steps dehydrating first, especially when comparing the neutron image of the partially dehydrated sample to the optical image of the same crystal. However, for a clearer view, in situ dehydration is required.

The observations made in Figures 7 and 8 can be coupled to the crystal structure of hydrated potassium carbonate. Using VESTA software and crystallographic data (AMCSD 0010365), the crystal structure was generated.

**Figure 9:**
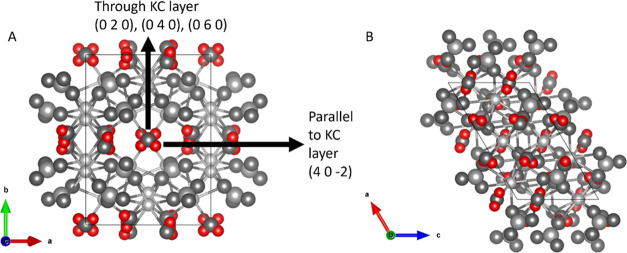
Crystal structures were generated using VESTA software and crystallographic data (AMCSD 0010365) along the *c* (A) and *b* (B) axes. The different water migration paths during hydration are given. The dense potassium carbonate layer is denoted as the KC layer. Potassium, carbon, and oxygen are displayed in gray scale, whereas hydrogen is displayed in red to emphasize the water positions. Miller indices are given in between brackets.

The generated unit cell for potassium carbonate sesquihydrate along the *c* and *b* axis is given in Figure 9A,9B, respectively. Figure 9A shows the placement of water molecules within the unit cell. The water molecules are “sandwiched” in between two dense potassium carbonate layers (KC layers). Upon removal of water molecules during dehydration, the water molecule can leave the crystal by migrating through the KC layer (along the *b* axis) or between the KC layers (parallel to the KC layer). Migration through the dense KC layer will be more difficult compared to migration parallel to the KC layers. As a result, water removal resulting from migration between the layers may, therefore, be preferential. Figure 9B shows the unit cell across the *b* axis, illustrating the dense structure of the KC layer.

**Figure 10:**
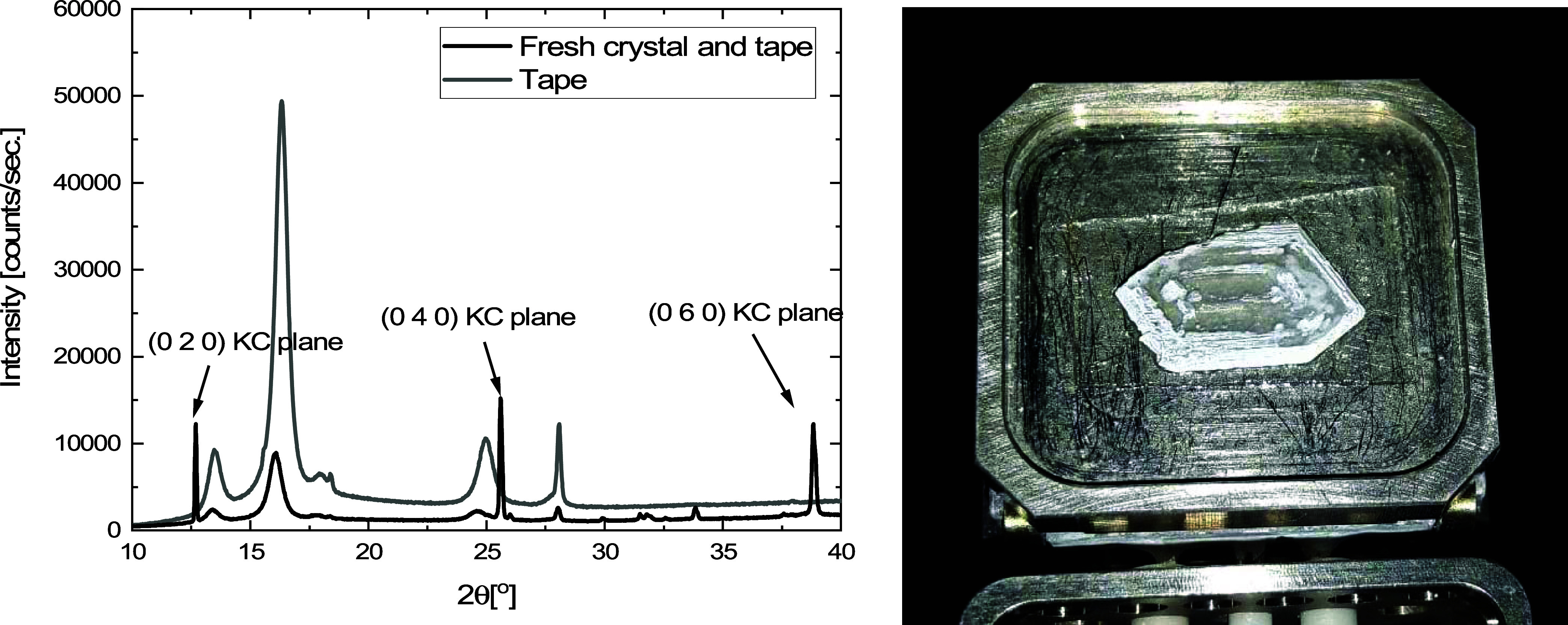
XRD measurement of a fresh single crystal, together with the identifiable crystallographic planes. The peaks originating from the dense potassium carbonate layer are denoted as the KC layer with Miller indices (*hkl*). The water plane with Miller indices represents the plane almost perpendicular to the KC plane. The photograph shows the placement of the single crystal in the XRD sample holder looking onto the (020), (040), and (060) planes.

The unit cell structures from Figure 9 indicate why single crystal dehydration starts at the edges. The water removed can leave a single crystal at these edges by migrating in between the KC layers, after which the dehydration moves inward from these edges. This is supported by the X-ray diffraction pattern of a single crystal (like that of Figures 7 and 8), which is shown in Figure 10. In this experiment, the single crystal was taped to the sample holder using double-sided tape to ensure a flat top surface. Therefore, the XRD measurement of the tape is included in Figure 10. Using the predicted powder diffraction pattern using VESTA software, the relevant crystallographic planes could be assigned. Since the only planes identifiable are the dense KC planes, the observations from microscopy can be directly linked to the crystal structure.

**Figure 11:**
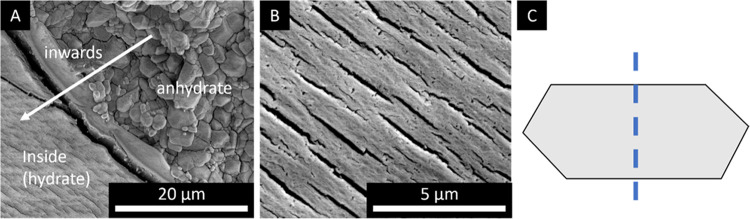
SEM images of a cleaved potassium carbonate sesquihydrate single crystal dehydrated for 6 h at 130 °C in a laboratory oven at the dehydration interface (A) and at within the hydrous phase (B). The fracture plane is denoted by the blue dashed line (C). Figure A is adapted with permission from.^25^ Copyright 2022 by Natalia Mazur.

The inward moving dehydration front is also visible in SEM images of a partially dehydrated potassium carbonate hydrate single crystal. Figure 11A shows the inward moving dehydration front from the edges of the single crystal toward the center. After dehydration, the morphology of the hydrous salt has changed from a solid crystal into an ensemble of micrometer-sized grains (Figure 11A). Similar observations have been made in larger K_2_CO_3_ particles.^9^

From Figure 11, it is visible that prior to dehydration, cracking within the hydrous phase occurs. The formed cracks are perpendicular to the movement of the front (Figure 9). The formed cracks facilitate the removal of water, after which the dehydration proceeds and the formation of micrometer-sized grains occurs. According to the orientation of the formed cracks with respect to the fracture plane and front direction, it is hypothesized that the cracks are formed in between the KC layers due to pressure built up of the escaping water.

### 3.7 Translating the Single Crystal Behavior toward Powder Samples

**Figure 12:**
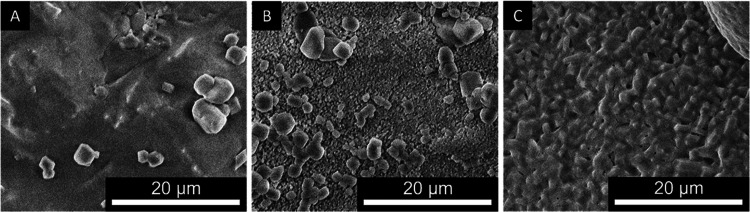
Powder SEM image of the fresh starting material (A), dehydrated material at 130 °C and 5 mbar (B), and rehydrated material at 25 °C and 5 mbar (C). The dehydration and rehydrating were performed inside the TGA furnace.

To link the observations on single crystals to powder. SEM images were taken of freshly hydrated, dehydrated, and rehydrated potassium carbonate powder. Three SEM images of the fresh starting material (Figure 12A), dehydrated material at 130 °C and 5 mbar (Figure 12B), and rehydrated material at 25 °C and 5 mbar (Figure 12C). The dehydration and rehydration were performed inside the TGA.

The fresh material shows a dense, nonporous, surface (Figure 12A). After dehydration (Figure 12B), a structure similar to that in the single crystal is observed (Figure 11). The solid structure has transformed into an assembly of small grains. After rehydration, this structure remains intact even though the grains have grown due to water uptake.

**Figure 13:**
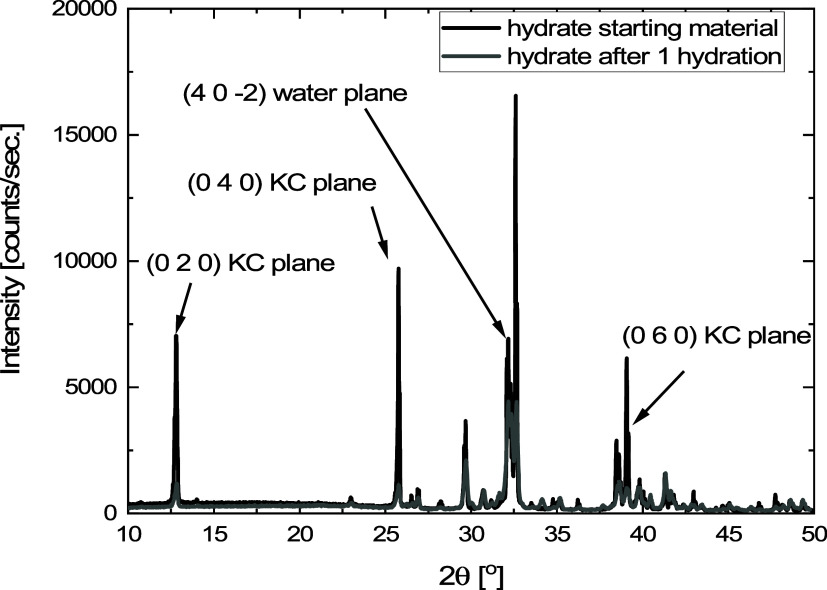
Powder XRD measurements of the fresh hydrated material (black line) and the same material after 1 in situ dehydration and hydration cycle (dehydrated at 160 °C and rehydrated at 25 °C, isobaric water vapor pressure of 5 mbar). The peaks originating from the dense potassium carbonate layer are denoted as the KC layer with Miller indices (hkl). The water plane with Miller indices represents the plane almost perpendicular to the KC plane.

An in situ hydration reaction was performed inside XRD to investigate the effect of cycling on the observed crystallographic planes. The powder XRD measurements of the fresh, hydrated material, and rehydrated material are given in Figure 13 (Dehydrated at 160 °C and rehydrated at 25 °C, isobaric water vapor pressure of 5 mbar). Using predicted powder diffractograms generated from crystallographic data (AMCSD 0010365) with VESTA software, relevant crystallographic planes were assigned.

**Figure 14:**
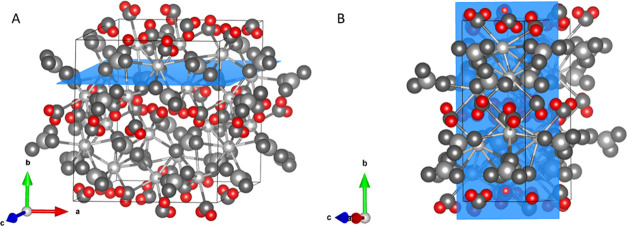
Crystal structures were generated by using VESTA software and crystallographic data (AMCSD 0010365). The blue shaded planes represent the (020), (040), (060) (A), and (402̅) planes (B) (*hkl*). Potassium, carbon, and oxygen are displayed in gray scale whereas hydrogen is displayed in red to emphasize the water positions.

Within the used scan range, 4 reflections are relevant: the 3 reflections corresponding to the dense KC planes, which are (020), (040), and (060) (Figure 14A), and the (402̅) reflection corresponding to the plane perpendicular to the KC layer, the plane responsible for easy water removal (Water plane in Figures 13 and 14B). The large peak to the right of the (402̅) peak corresponds to the (330) plane, which has a skewed/tilted orientation rendering it not suitable to describe the observations. Figures with the crystallographic plane and calculated versus experimental pattern are given in Supporting Information S1.5.

From the peak intensity of the assigned peaks in the XRD diffractograms, we infer that there are more dense KC network planes compared with the water planes in the case of the fresh material. After a single hydration, the reflections of the dense KC planes are reduced, while the reflection of the water plane is still present (Figure 13). The pattern of rehydrated K_2_CO_3_ resembles the ideal calculated powder based on the published crystallographic data more than the diffraction pattern measured from the pristine material. The decrease of KC planes and increase in water planes originate from the structural change during dehydration and consecutive hydration. Furthermore, using the Scherrer formula, we have calculated the average size of primary scattering domains in the pristine material to be 1039 ± 281 Å. After single dehydration and rehydration, the scattering domain size decreases to 549 ± 181 Å. The Miller indices of the used reflections are given in Supporting Information S1.6.

It was confirmed that during dehydration no amorphization occurred, and dehydration was fully completed by measuring the XRD diffractogram (in situ) of de anhydrous material. It was observed that all hydrate crystallographic planes disappeared, whereas the relevant anhydrous planes appeared. Furthermore, no amorphization was observed. For the XRD diffractogram, the reader is referred to Supporting Information S1.7.

**Figure 15:**
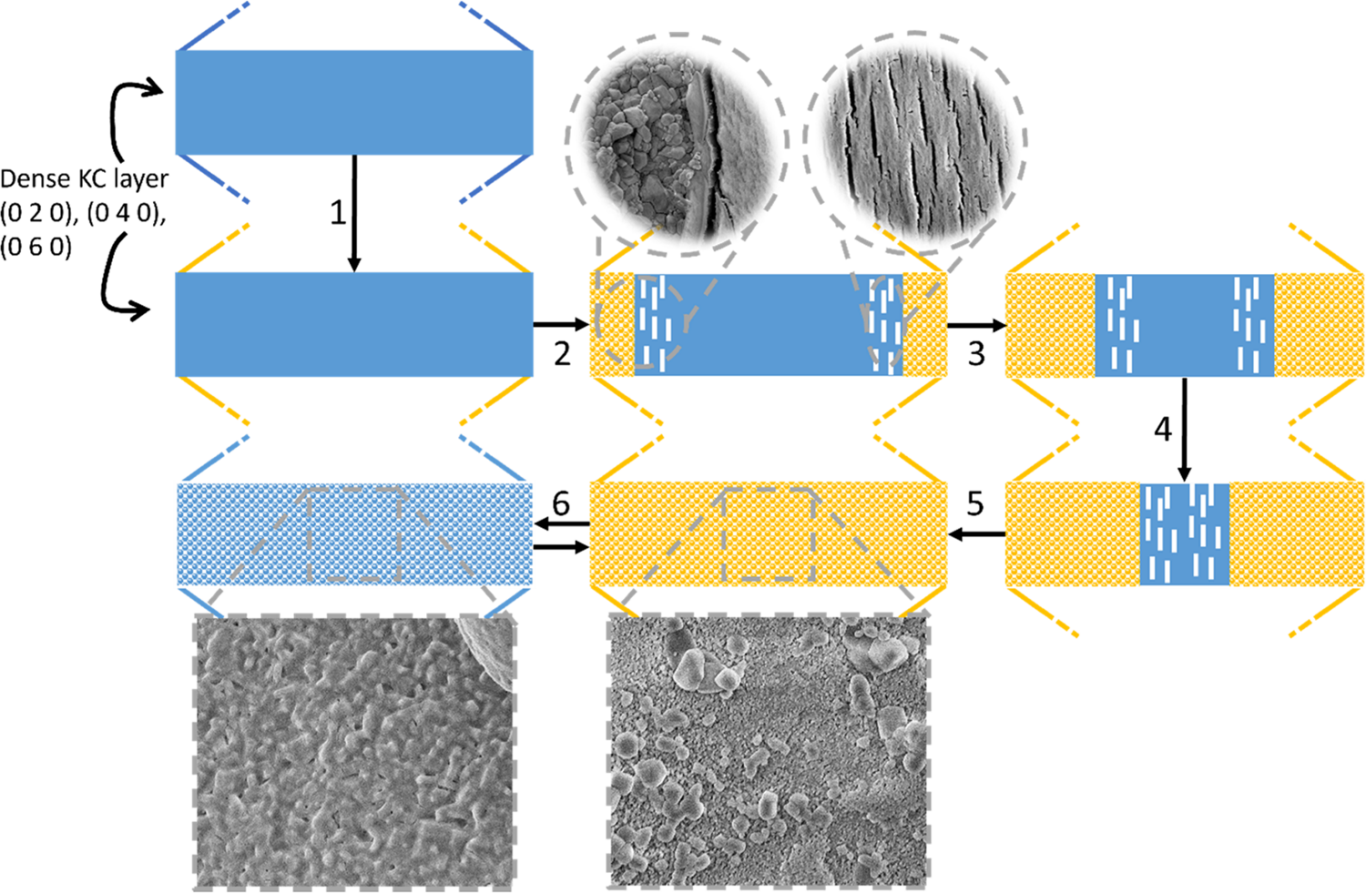
Schematic illustration of dehydration and hydration cycles starting from pristine hydrous material. The blue represents hydrous salt, and the orange represents anhydrous salt. The crack formation is indicated with the white lines. The gray dashed lines indicate the area to which the SEM images correspond to. The first solid blue image represents the hydrous pristine material with the dense KC layers oriented across the left and right border. The dashed lines indicate the continuation of the crystal. During step 1, the edges start to dehydrate first after which in step two the inside of the crystal also starts to dehydrate. Crack formation occurs prior to the dehydration front. As the front moves inward more hydrous material is transformed into micrometer-sized anhydrous grains (steps 3, 4, and 5). After rehydration the material cycles between the assembly of micrometer hydrous and anhydrous grains (step 6).

Dehydration of the pristine material mainly occurs through the dense KC layer, as this is the predominant crystallographic plane, resulting in a single process dehydration. During the first dehydration, crack formation occurs, and the pristine material is converted into an anhydrous assembly of micrometer-sized grains. During rehydration, it is observed that, predominantly, the water planes are reformed, whereas the dense KC layer reforms to a lesser extent. This facilitates water removal during dehydration, and dehydration now occurs as 2 events. Water removal is parallel to the KC planes, starting at lower temperatures with lower apparent activation energies, and water removal through the residual KC planes, starting at higher temperatures with higher apparent activation energies. A schematic summary is given in Figure 15.

## 4 Conclusions

In this work, the different processes during K_2_CO_3_·1.5H_2_O dehydration are investigated. It is found that the onset points for dehydration during dehydration event 1 are found at higher temperatures as what is expected from the literature. Also, this dehydration was found to occur as a single process (called process II). The onset points of consecutive dehydrations match with the available literature revealing two processes (process I and II).

It was found that the two processes have different activation energies starting with a low value toward a high value. With higher cycles of dehydration, the activation energy progresses toward a single value, indicating a single process at higher cycles. Optical microscopy of single crystal dehydration showed two different processes, dehydration at the edges followed by an inward moving dehydration front. The anhydrous material was found to undergo morphological restructuring into an assembly of small grains.

The observed morphological changes were found to result in two dehydration processes in the powder material. Crystallographic data and powder XRD showed two pathways for water removal through a dense layer and parallel to these layers. After the first cycle, many of these water planes, compared to dense KC planes, become available, resulting in double dehydration behavior.

The observations from this work may contribute to material design, as this gives insight into the effect of morphological changes and material structure on the dehydration behavior.

## Supporting Information Available

An example measurement sequence; cyclic hydration rates versus temperature; SEM image of cycled anhydrous powder; procedure for determining onset points; crystal structure and predicted XRD diffractogram of potassium carbonate sesquihydrate; Miller indices used with the Scherrer formula; and PXRD of anhydrous potassium carbonate (PDF)

## Abbreviations

KPI’s: key performance indicators
TGA: thermogravimetric analysis
RH: relative humidity
DSC: differential scanning calorimetry
PXRD: powder X-ray diffraction
SEM: scanning electron microscopy
MSZ: metastable zone
