# Supplementary material for: Elucidating the Dehydration Pathways of K2CO3·1.5H2O
Source: Cryst Growth Des. 2024 Mar 4;24(6):2493–504. doi: 10.1021/acs.cgd.3c01484 (PMC10958447; doi:10.1021/acs.cgd.3c01484)
Supplement: Supplementary file 1 — cg3c01484_si_001.pdf [file cg3c01484_si_001.pdf]

Elucidating the dehydration pathways of $K_2CO_3 \cdot 1.5H_2O$

Joey Aarts[a, b], Natalia Mazur[a,c], Hartmut R. Fischer[c], Olaf C. G. Adan[b, c], Henk P. Huinink[a, b, *]

a Eindhoven Institute of Renewable Energy Systems, Eindhoven University of Technology, PO Box 513, Eindhoven 5600 MB, the Netherlands
b Transport in Permeable Media group, Department of Applied Physics, Eindhoven University of Technology, PO Box 513, Eindhoven 5600 MB, the Netherlands
c TNO Materials Solution, PO Box 6235, High Tech Campus 25 Eindhoven 5600 HE, the Netherlands
* Corresponding author

S1.1. Example measurement sequence 5 mbar

Figure 1 shows an example sequence for an isobaric cyclic measurement at 5 mbar. For other vapor pressures the used sequence is the same apart from a different lowest temperature, scan rate, or number of cycles. First the material is kept at the hydration temperature to ensure that the material is fully hydrous at the start (flat weight line). After the temperature ramp the sample is kept at the highest temperature longer as in all consecutive cycles to ensure full dehydration and decarbonization. Afterwards, a temperature ramp is performed, and full hydration is performed at the lowest temperature.

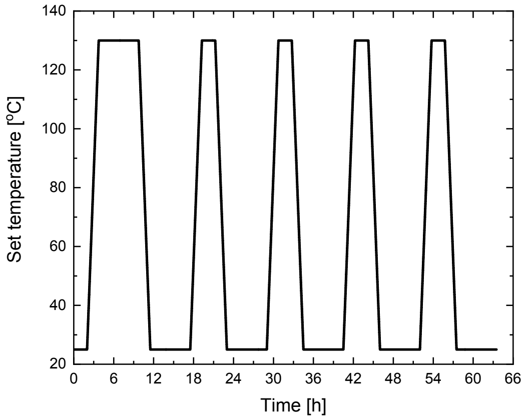

Figure 1. Example measurement sequence for isobaric measurements at 5 mbar.

S1.2. Hydration rate versus temperature at 5 mbar

Hydration rate versus sample temperature for the first 5 cycles.

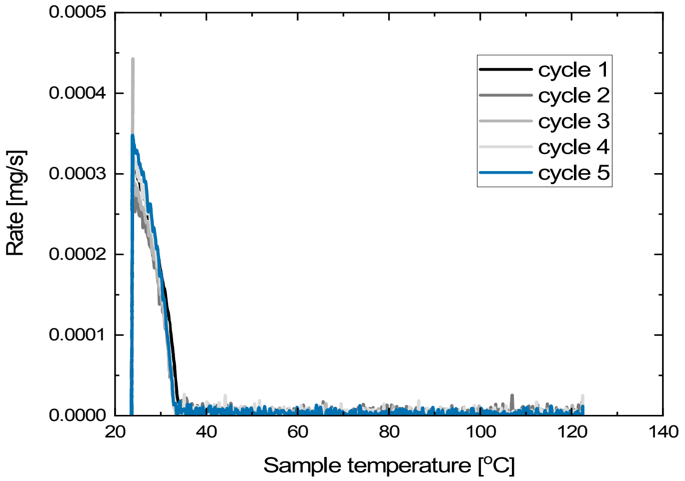

Figure 2. TGA cyclic rate curves versus sample temperature for 5 cycles of hydration at 5 mbar of water vapor pressure.

S1.3. SEM image of anhydrous powder after 11 dehydration events

SEM image of cycled powder using high vacuum (<10^{-4} mbar).

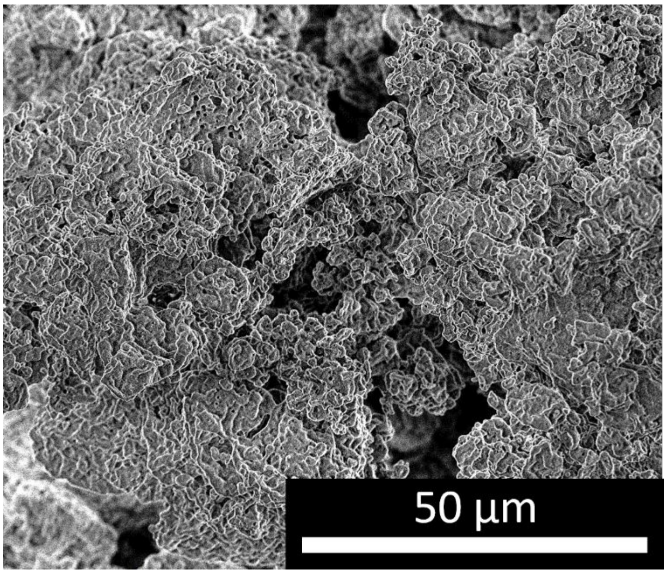

Figure 3. SEM image of anhydrous powder after 11 dehydration events.

S1.4. Determination of onset points

Onset points were determined using a linear fit (y=ax+b). The onset temperature (x) was determined by setting y=0. The linear fit was taken at the linear part of the rate curve. See Figure 4 for an example on how this was done for isobaric dehydration measurements at 5 mbar.

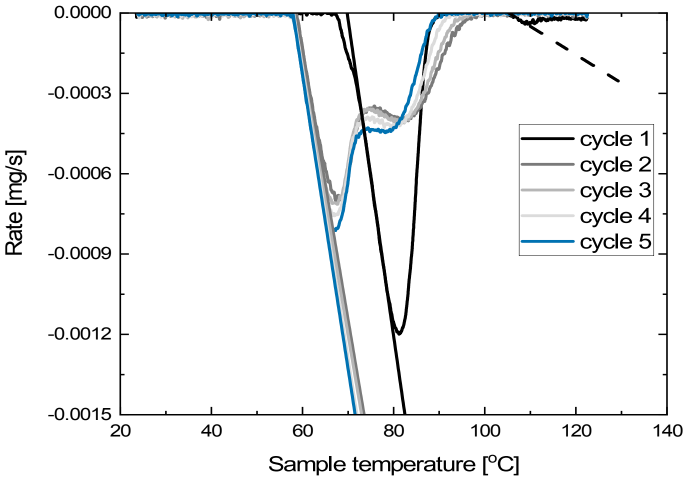

Figure 4. Example on how to determine the onset points for dehydration at isobaric conditions of 5 mbar. A linear fit was used to determine the onset points for dehydration (solid straight lines) and decarbonization (dashed line).

S1.5. (3 3 0) plane of potassium sesquihydrate and calculated pattern

Crystal structure generated using VESTA software together with the (3 3 0) plane.

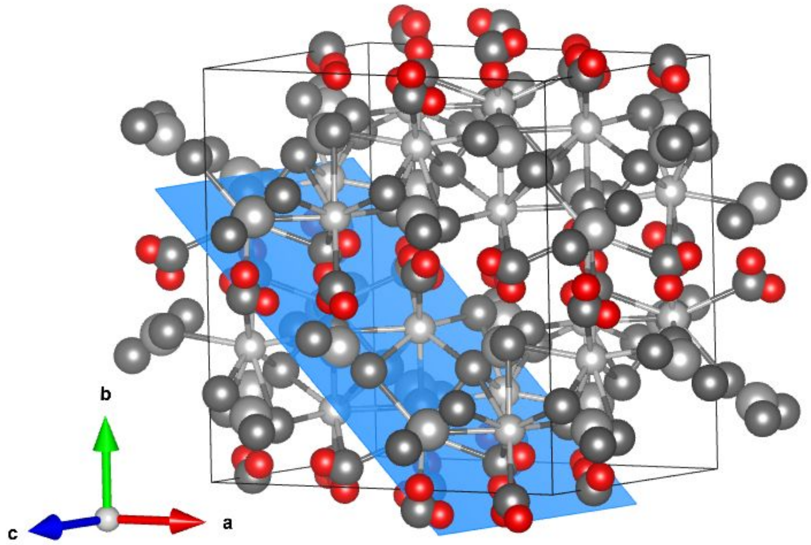

Figure 5. Crystal structure generated using VESTA software and crystallographic data (AMCSD 0010365). The blue shaded plane represents the (3 3 0) plane (h k l).

Below the calculated and experimental pattern for potassium carbonate sesquihydrate are given. The calculated pattern was generated using VESTA software and crystallographic data (AMCSD 0010365). The experimental pattern was measured using fresh material (Figure 13 in the manuscript). All data was normalized between 0 and 1.

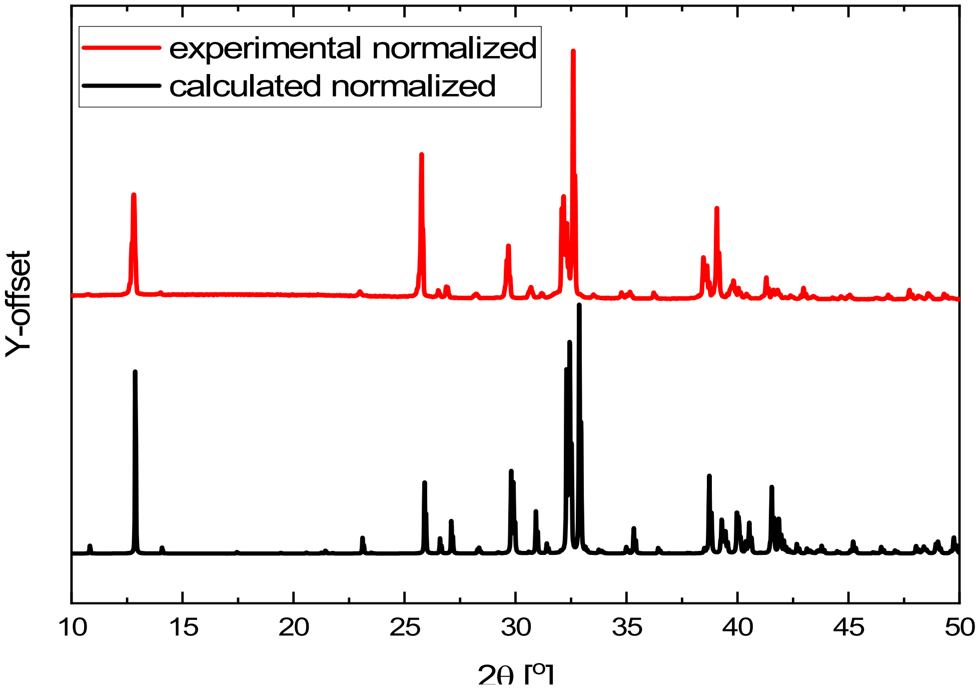

Figure 6. Calculated and experimental XRD diffractogram of potassium carbonate sesquihydrate. The calculated pattern was generated using VESTA software and crystallographic data (AMCSD 0010365). All data was normalized between 0 and 1.

S1.6. Miller indices used with the Scherrer formula

Miller indices (h k l) identified by PDXL software by Rigaku used with the Scherrer formula for the fresh material and after 1 hydration.

Table 1. Miller indices (h k l) used with the Scherrer formula for the fresh material and after 1 hydration.

Hydrate starting material	After 1 hydration
(0 2 0)	(0 2 0)
(1 1 -1)	(1 3 0)
(1 3 0)	(1 3 -1)
(1 3 -1)	(0 4 0)
(0 4 0)	(1 1 -2)
(1 1 -2)	(3 1 0)
(1 3 1)	(1 3 1)
(0 4 1)	(0 4 1)
(2 2 1)	(2 2 1)
(0 2 2)	(2 4 0)
(4 0 -2)	(4 0 -2)
(1 3 -2)	(1 3 -2)
(1 5 0)	(3 3 0)
(4 2 -2)	(4 2 -2)
(1 1 2)	(1 1 2)
(2 4 -2)	(2 4 -2)
(5 1 -2)	(4 2 0)
(3 5 -1)	(2 4 1)
(2 2 -3)	(5 1 -2)

(3 3 1)	(3 5 -1)
(4 4 -2)	(4 4 -1)
(1 5 -2)	(2 2 -3)
(3 5 0)	(3 3 1)
(3 3 -3)	(4 4 -2)
(5 3 -2)	(1 5 -2)
(5 1 -3)	(2 6 -1)
(1 3 -3)	(3 3 -3)
(0 2 3)	(5 3 -2)
(1 5 2)	(5 1 -3)
(3 5 1)	(5 1 0)
(6 2 -1)	(1 3 -3)
	(6 0 -2)
	(0 2 3)
	(1 7 0)
	(1 7 -1)
	(1 5 2)
	(2 6 1)
	(3 5 1)

S1.7. PXRD of anhydrous potassium carbonate

PXRD of in situ dehydrated potassium carbonate sesquihydrate at 160 °C.

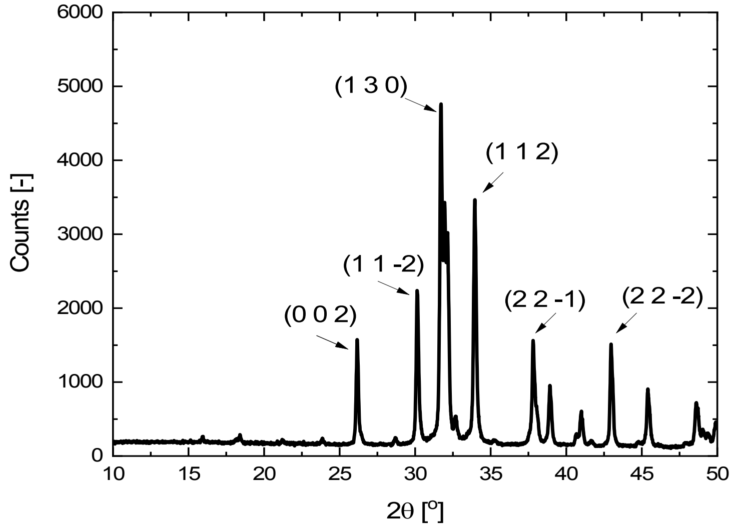

Figure 7. Powder XRD measurement of anhydrous potassium carbonate (in situ dehydrated sesquihydrate at 160 °C). Using predicted powder diffractograms generated from crystallographic data (COD 9009644) with VESTA software, relevant crystallographic planes were assigned.
